# Multi-locus sequence typing of *Mycoplasma bovis* to assess its genetic diversity from 2009 to 2018 in Ningxia Hui Autonomous Region, China

**DOI:** 10.1186/s12917-020-02668-x

**Published:** 2020-11-23

**Authors:** Yanan Guo, Haifeng Luo, Shuqiang Guo, Yuanyuan Lei, Yong Li, Shenghu He

**Affiliations:** 1 Department of Veterinary Clinical Sciences, School of Agriculture, Ningxia University, 750021 Yinchuan, Ningxia, China; 2 Technology Center Department, Yinchuan Customs, 750000 Yinchuan, Ningxia China; 3 Animal Disease Prevention and Control Center of Yan’an, 716000 Yan’an, Shaanxi China; 4 Department of Life Science and Technology, Ningxia Polytechnic College, 750021 Yinchuan, Ningxia China

**Keywords:** Multi-locus sequence typing, *Mycoplasma bovis*, genetic diversity, Ningxia Hui Autonomous Region

## Abstract

**Background:**

*Mycoplasma bovis* (*M. bovis*) is a highly contagious cattle pathogen spreading worldwide and especially in Ningxia Hui Autonomous Region in China.

**Results:**

Two types of ST, ST10and ST134, were identified in Ningxia Hui Autonomous Region. Thirty-seven strains belonged to ST10 and 28 strains belonged to ST134. ST134 was a new ST and first found in 2009 and was only widely distributed in Ningxia Hui Autonomous Region at present. The *M. bovis* ST10 was widely spread in many provinces in China and was widespread in Ningxia Hui Autonomous Region since 2010. It is speculated that the prevalence of *M. bovis* ST10 in Ningxia Hui Autonomous Region began in 2010.

**Conclusions:**

This study is the first report on the genetic diversity of *M. bovis* from 2009 to 2018 in Ningxia Hui Autonomous Region and provides the epidemiological information. These results may help further our understanding of the evolution of *M. bovis* and provide information that may be useful for the development of novel vaccines.

## Background

*Mycoplasma bovis* (*M. bovis*) is an important pathogen causing severe pneumonia, mastitis, and arthritis in the world. Especially, pneumonia caused by *M. bovis* has high morbidity and mortality. It is becoming one of the most widely recognized pathogens in the world[1, 2]. The pathogen is highly contagious and can spread rapidly throughout the herd. *M. bovis* can not only cause pneumonia, mastitis, arthritis, and otitis but also induce postpartum infection of the uterus with a mortality rate of 80%[3]. Since there are no effective vaccines and drugs to prevent and cure the disease caused by the pathogen, the incidence of the disease is on the rise[4, 5].

With the completion of whole-genome sequencing of *M. bovis*, a variety of highly repeatable molecular typing methods have been developed for molecular epidemiology and population structure research, including arbitrarily primed PCR (AP-PCR), random amplified polymorphic DNA (RAPD)[6], amplified fragment length polymorphism (AFLP)[7], pulsed-field gel electrophoresis (PFGE)[8], insertion sequence (IS)[9], variable number of tandem repeats (VNTR)[10, 11], multiple-locus variable-number tandem repeat (MLVA)[10] and multi-locus sequence typing (MLST)[5]. Although AP-PCR, RAPD, AFLP, and PFGE methods can obtain a large amount of genetic information, they are subjective in the analysis of DNA fragments and require special equipment[5], so it is difficult to establish a standardized method.

MLST is a rapidly developing molecular biology analysis method with high resolution in recent years. It is suitable for both molecular epidemiological studies and molecular advancement studies. The MLST method compares the nucleic acid sequences of the core fragments of several housekeeping genes and then compares the diversity of the alleles of the strains. Different strains correspond to different sequence types (ST)[4]. Through the STs of *M. bovis* pathogens can be used to understand the genetic diversity, population structure, and evolutionary trend, which will be beneficial to the control of *M. bovis* and the development of vaccines, as well as providing a theoretical basis for the prevention and control of *M. bovis*[4]. MLST is a typing technique based on seven housekeeping genes of *M. bovis* to study the genetic diversity, population structure, and evolutionary trend of *M. bovis*, including alcohol dehydrogenase-1 (*adh-1*), glutamate tRNA ligase (*gltX*), glycerol-3-phosphate dehydrogenase (*gpsA)*, DNA gyrase subunit B (*gyrB*), phosphate acetyltransferase-2 (*pta-2*), thymidine kinase (*tdk*) and transketolase (*tkt*)[12]. The MLST data were used to populate a newly created and publicly available database (www.pubmlst.org/mbovis) intended to serve as a tool for epidemiologic studies and further investigating the population structure of *M. bovis* [5]. MLST is a powerful, scalable, and highly standardized method that makes it easy to clearly distinguish housekeeping genes among different strains[13].

Ningxia Hui Autonomous Region is one of the most important raising regions for cow and beef cattle in China[14]. The feeding level of bovine is currently ranked second in China. In 2009, our team isolated *M. bovis* for the first time from the lung of cows in Ningxia Hui Autonomous Region. From 2009 to 2018, a total of 65 strains of *M. bovis* were obtained from samples of lung, synovial fluid, nasal swab, and milk in Ningxia Hui Autonomous Region. However, there is no related study on the molecular epidemiology and population structure of *M. bovis* in Ningxia Hui Autonomous Region. In this study, The MLST method was used to classify 65 isolates from different cities of Ningxia Hui Autonomous Region from 2009 to 2018, aiming to investigate the population structure of *M. bovis*[2, 5] and to explore the evolutionary relationship of Ningxia Hui Autonomous Region isolates with Chinese isolates and global isolates, which will lay a foundation for further prevention and control of *M. bovis* in the world.

## Results

### Strains identification

Sixty-seven isolates were identified as *M. bovis* by PCR amplification using *16S rRNA* and *uvrC* primers and sequencing of amplified products.

### MLST analysis of *M. bovis* isolates

.

A total of 3 STs were identified among the 67 strains of *M. bovis*. Among the 65 isolates from Ningxia Hui Autonomous Region, 37 isolates belonged to ST10 and 28 isolates belonged to ST134. The HB0801 and PG45 isolates belonged to ST10 and ST17, respectively (Table 1).

**Table 1:** M. bovis strains used in this study and their STs

Sample ID	Year of isolation	Origin	Host	Sample type	Clinical status	adh1	gltX	gpsA	gyrB	pta2	tdk	tkt	ST	Source
PG45	1961	American	Dairy cow	Milk	Clinical mastitis	3	2	4	2	1	3	2	17	This study
HB0801	2008	Hubei	Beef cattle	Lung	Pneumonia	4	3	2	3	5	3	4	10	This study
Hubei-1	2008	Hubei	Cattle	Lung	Pneumonia	4	3	2	3	5	4	4	26	MLST web
EZ-8-NHD0962	2008	Hubei	Bovine	Lung	Pneumonia	4	5	2	3	5	3	4	32	MLST web
CQ-W70	2009	Chongqing	Bovine	Lung	Pneumonia	4	5	2	3	5	3	4	32	MLST web
EZ-2	2008	Hubei	Bovine	Lung	Pneumonia	4	3	2	12	5	3	4	43	MLST web
NHD0986	2008	Hunan	Bovine	Lung	Pneumonia	4	3	2	12	5	3	4	43	MLST web
NMH7	2018	Inner Mongolia	Bovine	Milk	Mastitis	10	3	6	13	21	6	10	173	MLST web
NMH03	2018	Inner Mongolia	Bovine	Milk	Mastitis	10	3	6	13	21	6	10	173	MLST web
HBHS01	2018	Hubei	Bovine	Milk	Mastitis	4	3	2	3	5	7	4	172	MLST web
Shaanxi04	2018	Shaanxi	Bovine	Milk	Mastitis	4	3	2	3	5	7	4	172	MLST web
NX001	2009	Wuzhong	Dairy cow	Milk	Clinical Mastitis	4	3	2	3	17	3	4	134	This study
NX002	2010	Wuzhong	Dairy cow	Lung	Pneumonia	4	3	2	3	5	3	4	10	This study
NX003	2010	Wuzhong	Dairy calf	Joint fluid	Arthritis	4	3	2	3	5	3	4	10	This study
NX004	2010	Wuzhong	Dairy calf	Joint fluid	Arthritis	4	3	2	3	17	3	4	134	This study
NX005	2010	Wuzhong	Dairy cow	Nose Swab	Pneumonia	4	3	2	3	5	3	4	10	This study
NX006	2010	Shizuishan	Dairy cow	Nose Swab	Pneumonia	4	3	2	3	5	3	4	10	This study
NX007	2010	Wuzhong	Dairy calf	Joint fluid	Arthritis	4	3	2	3	5	3	4	10	This study
NX008	2011	Guyuan	Beef cattle	Lung	Pneumonia	4	3	2	3	17	3	4	134	This study
NX009	2011	Yinchuan	Dairy cow	Nose Swab	Pneumonia	4	3	2	3	5	3	4	10	This study
NX010	2011	Wuzhong	Dairy cow	Lung	Pneumonia	4	3	2	3	5	3	4	10	This study
NX011	2011	Yinchuan	Dairy cow	Nose Swab	Pneumonia	4	3	2	3	5	3	4	10	This study
NX012	2012	Wuzhong	Dairy cow	Nose Swab	Pneumonia	4	3	2	3	5	3	4	10	This study
NX013	2012	Wuzhong	Dairy cow	Nose Swab	Pneumonia	4	3	2	3	5	3	4	10	This study
NX014	2012	Guyuan	Beef cattle	Lung	Pneumonia	4	3	2	3	5	3	4	10	This study
NX015	2013	Wuzhong	Beef cattle	Lung	Pneumonia	4	3	2	3	5	3	4	10	This study
NX016	2013	Yinchuan	Dairy cow	Milk	Clinical mastitis	4	3	2	3	17	3	4	134	This study
NX017	2013	Shizuishan	Dairy cow	Milk	Clinical mastitis	4	3	2	3	17	3	4	134	This study
NX018	2013	Yinchuan	Dairy cow	Milk	Clinical mastitis	4	3	2	3	17	3	4	134	This study
NX019	2013	Yinchuan	Dairy cow	Milk	Clinical mastitis	4	3	2	3	17	3	4	134	This study
NX020	2013	Yinchuan	Dairy cow	Nose Swab	Pneumonia	4	3	2	3	5	3	4	10	This study
NX021	2013	Guyuan	Beef cattle	Lung	Pneumonia	4	3	2	3	5	3	4	10	This study
NX022	2013	Yinchuan	Dairy calf	Joint fluid	Arthritis	4	3	2	3	17	3	4	134	This study
NX023	2014	Yinchuan	Dairy cow	Nose Swab	Pneumonia	4	3	2	3	17	3	4	134	This study
NX024	2014	Yinchuan	Dairy cow	Milk	Clinical mastitis	4	3	2	3	5	3	4	10	This study
NX025	2014	Yinchuan	Dairy cow	Lung	Pneumonia	4	3	2	3	17	3	4	134	This study
NX026	2014	Yinchuan	Dairy cow	Nose Swab	Pneumonia	4	3	2	3	17	3	4	134	This study
NX027	2014	Yinchuan	Dairy cow	Lung	Pneumonia	4	3	2	3	17	3	4	134	This study
NX028	2014	Yinchuan	Dairy cow	Lung	Pneumonia	4	3	2	3	17	3	4	134	This study
NX029	2014	Shizuishan	Beef cattle	Lung	Pneumonia	4	3	2	3	5	3	4	10	This study
NX030	2014	Yinchuan	Dairy cow	Nose Swab	Pneumonia	4	3	2	3	5	3	4	10	This study
NX031	2014	Yinchuan	Dairy cow	Milk	Clinical mastitis	4	3	2	3	17	3	4	134	This study
NX032	2014	Yinchuan	Dairy cow	Milk	Clinical mastitis	4	3	2	3	17	3	4	134	This study
NX033	2015	Wuzhong	Dairy calf	Joint fluid	Arthritis	4	3	2	3	17	3	4	134	This study
NX034	2015	Wuzhong	Dairy calf	Joint fluid	Arthritis	4	3	2	3	17	3	4	134	This study
NX035	2015	Wuzhong	Dairy calf	Joint fluid	Arthritis	4	3	2	3	17	3	4	134	This study
NX036	2015	Wuzhong	Dairy calf	Joint fluid	Arthritis	4	3	2	3	17	3	4	134	This study
NX037	2015	Wuzhong	Dairy calf	Joint fluid	Arthritis	4	3	2	3	17	3	4	134	This study
NX038	2015	Shizuishan	Dairy cow	Milk	Clinical Mastitis	4	3	2	3	17	3	4	134	This study
NX039	2016	Guyuan	Beef cattle	Lung	Pneumonia	4	3	2	3	5	3	4	10	This study
NX040	2016	Guyuan	Beef cattle	Lung	Pneumonia	4	3	2	3	5	3	4	10	This study
NX041	2016	Guyuan	Beef cattle	Lung	Pneumonia	4	3	2	3	5	3	4	10	This study
NX042	2016	Guyuan	Beef cattle	Lung	Pneumonia	4	3	2	3	5	3	4	10	This study
NX043	2017	Yinchuan	Dairy calf	Joint fluid	Arthritis	4	3	2	3	5	3	4	10	This study
NX044	2017	Yinchuan	Dairy calf	Joint fluid	Arthritis	4	3	2	3	5	3	4	10	This study
NX045	2017	Yinchuan	Dairy calf	Joint fluid	Arthritis	4	3	2	3	5	3	4	10	This study
NX046	2018	Guyuan	Beef cattle	Lung	Pneumonia	4	3	2	3	5	3	4	10	This study
NX047	2018	Yinchuan	Dairy calf	Lung	Pneumonia	4	3	2	3	17	3	4	134	This study
NX048	2018	Yinchuan	Dairy calf	Lung	Pneumonia	4	3	2	3	17	3	4	134	This study
NX049	2018	Wuzhong	Dairy calf	Joint fluid	Arthritis	4	3	2	3	5	3	4	10	This study
NX050	2018	Wuzhong	Dairy calf	Joint fluid	Arthritis	4	3	2	3	5	3	4	10	This study
NX051	2018	Yinchuan	Dairy calf	Joint fluid	Arthritis	4	3	2	3	5	3	4	10	This study
NX052	2018	Yinchuan	Dairy calf	Joint fluid	Arthritis	4	3	2	3	5	3	4	10	This study
NX053	2018	Yinchuan	Dairy calf	Joint fluid	Arthritis	4	3	2	3	5	3	4	10	This study
NX054	2018	Yinchuan	Dairy calf	Joint fluid	Arthritis	4	3	2	3	5	3	4	10	This study
NX055	2018	Yinchuan	Dairy calf	Joint fluid	Arthritis	4	3	2	3	5	3	4	10	This study
NX056	2018	Yinchuan	Dairy calf	Joint fluid	Arthritis	4	3	2	3	5	3	4	10	This study
NX057	2018	Yinchuan	Dairy calf	Joint fluid	Arthritis	4	3	2	3	5	3	4	10	This study
NX058	2018	Yinchuan	Dairy calf	Joint fluid	Arthritis	4	3	2	3	5	3	4	10	This study
NX059	2018	Yinchuan	Dairy calf	Lung	Pneumonia	4	3	2	3	5	3	4	10	This study
NX060	2018	Wuzhong	Dairy calf	Lung	Pneumonia	4	3	2	3	5	3	4	10	This study
NX061	2018	Yinchuan	Dairy calf	Nose Swab	Pneumonia	4	3	2	3	17	3	4	134	This study
NX062	2018	Yinchuan	Dairy calf	Nose Swab	Pneumonia	4	3	2	3	17	3	4	134	This study
NX063	2018	Yinchuan	Dairy calf	Nose Swab	Pneumonia	4	3	2	3	17	3	4	134	This study
NX064	2018	Yinchuan	Dairy calf	Nose Swab	Pneumonia	4	3	2	3	17	3	4	134	This study
NX065	2018	Yinchuan	Dairy calf	Nose Swab	Pneumonia	4	3	2	3	17	3	4	134	This study

Wuzhong, Shizuishan, Yinchuan and Guyuan are different cities in Ningxia Hui Autonomous Region of China. Hubei, Chongqing, Hunan, Inner Mongolia, and Shaanxi are different provinces of China

According to Table 1, ST10 and ST134 strains could be isolated from samples of clinical mastitis (*n* = 9), arthritis (*n* = 22), and pneumonia (*n* = 34). The main clinical symptoms of calves were arthritis and pneumonia. The clinical symptoms of dairy cattle were mainly mastitis and pneumonia, while beef cattle were mainly pneumonia and arthritis.

The NX001 strain isolated from Ningxia Hui Autonomous Region for the first time in 2009 was ST134, and the NX002 strain isolated in 2010 was ST10. Since then, the ST10 and ST134 have been isolated from different lesions of bovine in different cities in Ningxia Hui Autonomous Region.

### Phylogenetic analysis

**Fig. 1:**
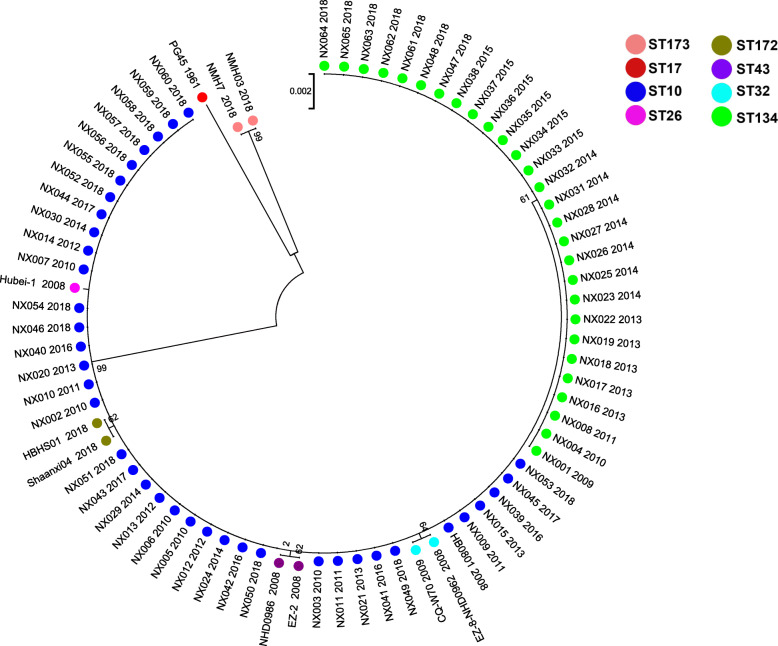
Phylogenetic tree constructed using maximum likelihood and Kimura 2-parameter model based on concatenated MLST sequence data

The phylogenetic tree constructed from the concatenated sequences of the seven target genes revealed two distinct lineages (Fig. 1). The ST173 and ST17 were in the same lineage. The other STs were in the other lineage including ST10, ST26, ST32, ST43, ST172, and the Ningxia Hui Autonomous Region isolates of ST134.

## Discussion

Molecular epidemiological studies are of great significance to reveal the population structure, genetic diversity, and prevalence of *Mycoplasma spp* [4, 15], which facilitates the formulation of effective prevention and control measures, including the development of vaccines and diagnostic methods[4, 16]. MLST studies performed on 44 strains from nine Chinese provinces from 2008 to 2014 showed that ST10, ST32, and ST43 were found in Hubei province (*n*= 25) and that ST10 was also found in Anhui (*n* = 1), Fujian (*n* = 2), Hunan (*n* = 1), Henan (*n* = 8), Inner Mongolia (*n* = 1), Jiangxi (*n* = 3), Guangzhou (*n* = 2), and Shandong (*n* = 1) province [4]. However, there are no reports about the MLST study of *M. bovis* in Ningxia Hui Autonomous Region. Therefore, this study is the first report on the molecular epidemiology of *M. bovis* from 2009 to 2018 in Ningxia Hui Autonomous Region.

At present, there are seven STs in *M. bovis* in China including ST10, ST26, ST32, ST43, ST134, ST172, and ST173[4]. ST10 is widely prevalent in all provinces reported in China, including Ningxia Hui Autonomous Region (*n* = 37, 56.9%) where are no MLST reports. After the ST10 strain was first isolated in 2010 in Ningxia Hui Autonomous Region, the ST10 of *M. bovis* could be isolated and identified every year. And it has been widely diffused in different cities in Ningxia Hui Autonomous Region. Interestingly, the ST10 strains have been reported that were widely distributed in American, Australia, and Israel[4]. Previously, the international spread of contagious bovine pleuropneumonia was shown to be linked to the movement of cattle[4, 17, 18]. So, it is a similar transfer probably that through international movement of cattle and domestic movement of cattle caused the widespread distribution of the *M. bovis* ST10 in China and even in Ningxia Hui Autonomous Region. The reason for the widespread prevalence of ST10 strain in Ningxia Hui Autonomous Region may be that the Ningxia Hui Autonomous Region government strongly supports the construction of large-scale cattle breeding parks. For their expansion, intensive cattle farms had to purchase cattle from different provinces of China and different countries in the world, but *M. bovis* was ignored, which led to the widespread presence of exogenous *M. bovis* in Ningxia Hui Autonomous Region. It is speculated that the prevalence of *M. bovis* ST10 in Ningxia Hui Autonomous Region began in 2010.

In 2009, our lab isolated *M. bovis* from the lung tissue of cows for the first time, which was ST134. From 2009 to 2018, ST134 (*n* = 28, 43.1%) was isolated and identified from different cities of Ningxia Hui Autonomous Region. However, it has not been identified in other provinces in China. Therefore, it confirmed that ST134 strains were closely related strains with the same origin in Ningxia Hui Autonomous Region, China, and has been widely distributed in Ningxia Hui Autonomous Region for many years.

To evaluate the evolutionary relationship of *M. bovis* between isolates from Ningxia Hui Autonomous Region and isolates from other provinces in China, a phylogenetic tree was constructed based on concatenated sequences. The evolutionary analysis showed that all ST10 strains were in the same lineage as ST26, ST32, ST43, ST172, including Ningxia Hui Autonomous Region ST134 strains. However, the ST173 strains were in the same lineage as the ST17 strain. The study results indicate that the *M. bovis* strains with STs different from ST173 and ST17 were closely related strains with the same origin.

## Conclusions

This study revealed the genetic diversity of *M. bovis* from 2009 to 2018 in Ningxia Hui Autonomous Region and provides epidemiological information. ST10 strains were widely prevalent in Ningxia Hui Autonomous Region as well as all provinces of China that have been reported, and ST134 strains were also widely distributed in Ningxia Hui Autonomous Region.

These results may help further our understanding of the evolution of *M. bovis* and provide information that may be useful for the development of novel vaccines.

## Methods

### Strains

The detailed information of sixty-seven strains of *M. bovis* was listed in Table 1, including the host, isolation site, geographical location, and the number of strains. Sixty-five strains came from different farms and were isolated from nasal swab, or milk, or joint fluid, or lung of *M. bovis*-infected cattle in Ningxia Hui Autonomous Region from 2009 to 2018. HB0801 strain was isolated from the lungs of beef cattle in Hubei Province in 2008 when *M. bovis* was first reported in China[19]. PG45 strain (ATCC 25,523) was donated by Professor Aizhen Guo of Huazhong Agricultural University of China.

### Cultivation and identification of *M. bovis*.

The *M. bovis* strains were cultured in PPLO broth(BD DifcoTM, US California)[4, 20]. *M. bovis* genomic DNA was extracted using a bacterial DNA extraction kit (Tiangen, Beijing, China)[21]. The*16S rRNA* gene[22] and *uvrC* gene[23] were amplificated using two pairs of primers (Table 2). The PCR reaction mixture was 50 µL (5 µL 10 × PCR buffer, 4 µL dNTP mixture, 0.25 µL rTaq, 2 µL primers, 1 µL DNA, 37.75µL ddH_2_O). The reaction mixture was incubated at 95 ℃ for 2 min, 35 cycles of 95 ℃ for 30 s, 50 ℃ for 20 s and 72 ℃ for 2 min, then a final incubation at 72 ℃ for 8 min[22, 23].

**Table 2:** Primers used for identification and amplification of MLST loci of *M. bovis*

Name	Sequence	Amplicon size (bp)
*16S rRNA* forward	5’-GAA TTC CGA GAG TTT GAT CCT GGC T-3’	1517
*16S rRNA* reverse	5’-AAG CTT GAG GTA ATC CAT CCC CAC GTT C-3’	
*uvrC* forward	5’-GAA TTC AAT GTG TCT ACT AGT CCT GG -3’	1620
*uvrC* reverse	5’-AAG CTT AGC GTC ATA GAT TTT TGC ATA-3’	
*adh-1* forward	5’- GGA GTA ACT AGT TAC AAA GCA CTT A -3’	546
*adh-1* reverse	5’- TGC TAG TTG TTC AAA CAC GT -3’	
*gltX* forward	5’- TGG TGA GTA TTC AAT AAG GT-3’	530
*gltX* reverse	5’- GTT TTG AGA ATC ATT GCA − 3’	
*gpsA* forward	5’- AAA ATG TGA GGA ATT GAT CA -3’	521
*gpsA* reverse	5’- CCA ATT CCA ATT GCT AAA AC -3’	
*gyrB* forward	5’- AGC TTG CTA ATT GCA CCA − 3’	678
*gyrB* reverse	5’- TAT TTT GAA CAA ATT TTG CAT − 3’	
*pta-2* forward	5’- AAT TCG TAA TGG CAA AGA AG -3’	490
*pta-2* reverse	5’- CTT AGC TTT TCT TAC ATT TAG GT -3’	
*tdk* forward	5’ –ATG TAT TTA AAA AGT GGA TTA GG -3’	572
*tdk* reverse	5’- TAT CTC ATA GCT TTT TTA GC -3’	
*tkt* forward	5’- CCA ACT TAT ATT ATG GTG CA -3’	533
*tkt* reverse	5’- CCA CCA TAT AAA TTA ATG CC -3’	

### Multi-locus sequence typing

The genes of 67 isolates of *M. bovis* were amplified by PCR using MLST scheme (*adh-1*, *gltX*, *gpsA*, *gyrB*, *pta-2*, *tdk* and *tkt*)[4, 5] (Table 2). The PCR reaction mixture was 50 µL (5 µL 10 × PCR buffer, 4 µL dNTP mixture, 0.25 µL rTaq, 2 µL primers, 1 µL DNA, 37.75µL ddH_2_O). The reaction mixture was incubated at 95 ℃ for 2 min, 35 cycles of 95 ℃ for 30 s, 55 ℃ for 30 s and 72 ℃ for 1 min, then a final incubation at 72 ℃ for 5 min[5]. The PCR amplification products were sent to Shanghai Bioengineering Co., Ltd for sequencing. The assembly sequences were aligned by the http://pubmlst.org/mbovis/ database to obtain the allele number and STs.

### Phylogenetic analysis

Seven gene sequences of 65 strains of *M. bovis* in Ningxia Hui Autonomous Region and the strains of different STs in China and PG45 reference strains were concatenated. A phylogenetic tree was constructed from concatenated sequences. The evolutionary history was inferred by using the Maximum Likelihood method based on the Kimura 2-parameter model[3] of MEGA 10.0. Initial tree(s) for the heuristic search was obtained automatically by applying Neighbor-Join and BioNJ algorithms to a matrix of pairwise distances estimated using the Maximum Composite Likelihood (MCL) approach and then selecting the topology with superior log likelihood value[3].

## Data Availability

The datasets generated and/or analysed during the current study are available in the National Center for Biotechnology Information repository (MW19432 - MW194385, MW194386 - MW194450, MW194451 - MW194515, MW194516 - MW194580, MW194581 - MW194645, MW194646 - MW194710, MW194711 - MW194775).

## Abbreviations

adh-1: Alcohol dehydrogenase-1
AFLP: Amplified fragment length polymorphism
AP-PCR: Arbitrarily primed PCR
ddH2O: Doble distilled water
DNA: Deoxyribonucleic acid
gltX: Glutamate tRNA ligase
gpsA: Glycerol-3-phosphate dehydrogenase
gyrB: DNA gyrase subunit B
IS: Insertion sequence
M. bovis: Mycoplasma bovis
MLST: Multi-locus sequence typing
PCR: Polymerase chain reaction
PFGE: Pulsed-field gel electrophoresis
PPLO: Pleuropneumonia-like organisms
pta-2: Phosphate acetyltransferase-2
RAPD: Random amplified polymorphic DNA
RNA: Ribosomal ribonucleic acid
ST: Sequence types
tdk: Thymidine kinase
tkt: Transketolase
VNTR: Variable number of tandem repeats
